# The formulation of an individualized nursing plan for elderly patients with acute cerebral infarction based on comfort nursing intervention and its influence on patient comfort

**DOI:** 10.3389/fneur.2026.1667931

**Published:** 2026-04-01

**Authors:** Ruiying Ding, Zhonghong Guo, Rongming Ding

**Affiliations:** 1 Department of Geriatrics, Ganzhou People's Hospital, Ganzhou, Jiangxi, China; 2 Department of Nursing, Ganzhou Maternal and Child Health Care Hospital, Ganzhou, Jiangxi, China; 3 Department of Cardiovascular, Ganzhou People's Hospital, Ganzhou, Jiangxi, China

**Keywords:** acute cerebral infarction, comfort nursing intervention, individual nursing, nursing, stroke

## Abstract

**Objective:**

To explore the effectiveness of comfort nursing intervention in the care of elderly patients with acute cerebral infarction to improve patient comfort and quality of life.

**Method:**

In this study, 120 elderly patients with acute cerebral infarction were randomly divided into an experimental group and a control group. The experimental group was given individualized nursing intervention based on comfort nursing intervention, including physical, psychological, social and environmental nursing. The control group received routine nursing measures. The National Institutes of Health Stroke Scale, Fugl-Meyer Assessment of Motor Function Scale, Activities of Daily Living Assessment Scale, Newcastle Nursing Satisfaction Scale and Hamilton Anxiety Scale were used to evaluate the degree of neurological deficits, motor function recovery, self-care ability of daily life, satisfaction with nursing services and anxiety level of the two groups of patients. The data were analyzed using SPSS software.

**Result:**

The effective implementation of comfort nursing intervention significantly improved the neurological function, motor ability, activities of daily living and nursing satisfaction of elderly patients with acute cerebral infarction, and reduced the level of anxiety (*p* < 0.01), and the overall satisfaction was significantly improved.

**Conclusion:**

The effective implementation of comfort nursing intervention significantly improved the comfort and quality of life of elderly patients with acute cerebral infarction, indicating that the application of this theory in clinical nursing is of great value.

## 1 Introduction

Acute cerebral infarction, commonly known as stroke, is due to the interruption of blood supply to the brain, resulting in brain tissue hypoxia and undernutrition, which leads to local neurological dysfunction (1, 2). Its pathogenesis is mainly related to vascular obstruction, and the common causes include thrombosis and embolism (3, 4). Elderly people are particularly prone to such diseases due to physiological decline and many basic diseases (5). Stroke is one of the leading causes of death and disability worldwide. The morbidity and mortality of the elderly population are considerably higher than in other age groups (6, 7). With the acceleration of global aging, the incidence of acute cerebral infarction in elderly people continues to rise; this has become a problem in the field of public health that needs to be resolved urgently (8).

Scientific nursing programmes play a vital role in the treatment and rehabilitation of acute cerebral infarction. Effective nursing can not only improve the patient's physical condition and promote the recovery of physical function but also considerably improve the patient's psychological state and quality of life (9). Individualized rehabilitation plans can formulate appropriate rehabilitation goals and strategies according to the specific needs of patients, thus maximizing the therapeutic effect. In addition, scientific nursing can reduce the psychological burden of patients and their families and help them better cope with the challenges posed by the disease (10). Therefore, strengthening the scientific nursing of patients with acute cerebral infarction not only helps to improve the survival rate and quality of life of patients but also reduces the cost and burden of nursing for society.

Comfort care theory emphasizes the creation of a comprehensive and comfortable treatment and nursing environment for patients, aiming to improve their overall comfort through physiological, psychological and social support (11, 12). The core aims of this theory are to alleviate the patient's discomfort, relieve negative emotions and enable them to obtain comprehensive physical and mental relaxation and support during the rehabilitation process. At the physiological level, nursing staff can improve the comfort of patients by providing appropriate environmental regulation (e.g., appropriate room temperature, lighting and a quiet nursing space), pain management (e.g., timely pain assessment and drug intervention) and individualized nutritional support. A good nursing environment can reduce external interference, providing patients with better quality rest and promoting the recovery of physical function. At the psychological level, nursing staff can offer emotional support to help patients express their emotions and reduce their psychological burden by establishing a good communication and trust relationship. At the same time, the use of relaxation techniques, music therapy and other methods can also effectively reduce the anxiety and tension of patients and improve their quality of life. Social support is also an important part of comfort care. By providing family education and support, encouraging family members to participate in the nursing process can not only enhance the patient's sense of belonging but also improve their psychological state and promote rehabilitation (13, 14). Therefore, comfort nursing intervention in the care of patients with acute cerebral infarction can effectively promote the recovery of physical function, improve psychological comfort and create a comprehensive support environment.

The purpose of this study is to explore the effect of individualized nursing programmes based on comfort nursing intervention on the comfort and overall recovery of elderly patients with acute cerebral infarction, thus providing a more scientific basis for clinical nursing. Through the implementation of individualized nursing programmes based on comfort care theory, this study systematically evaluates the effectiveness of nursing interventions in improving patients' physical comfort, reducing psychological burden and enhancing social support. In addition, the results of the study provide an important reference for future nursing practice and related policy formulation. Through empirical data, nursing staff and managers can understand more deeply the importance of comfort care, thus promoting the innovation and development of nursing modes.

## 2 Research participants and methods

### 2.1 Research participants

In this study, 120 elderly patients with acute cerebral infarction were randomly divided into an experimental group and a control group. The experimental group received individualized nursing programmes based on comfort nursing intervention, whereas the control group received routine nursing. The study collected general information of patients, including age, gender, marital status, body mass index (BMI), underlying diseases (e.g., hypertension, diabetes, dyslipidaemia) and other information for subsequent analysis and comparison. No participants dropped out during the study period.

The inclusion criteria were as follows: (1) aged ≥60 years, in line with the definition of elderly people; (2) diagnosed as acute cerebral infarction and participated in the study within 72 h after admission; (3) understood the research content, participated voluntarily and signed informed consent; (4) had cognitive ability and could perform effective communication and cooperation with nursing; and (5) had no serious complications (e.g., severe heart disease, renal failure) and could accept routine nursing and comfort nursing intervention.

The exclusion criteria were as follows: (1) received other related intervention studies in the past 6 months; (2) serious mental illness, cognitive impairment or inability to understand the research content; (3) serious complications (e.g., cerebral hemorrhage, infection) during the study period that required transferral to the intensive care unit; (4) refusal to participate in, or withdrawal from, the study for other reasons (e.g., family factors, personal wishes); and (5) other diseases that could affect the results of the study (e.g., malignant tumors, severe lung diseases).

### 2.2 Nursing methods

Routine nursing programme: (1) regular monitoring of the patient's heart rate, blood pressure, respiratory rate and body temperature; (2) timely detection of abnormalities; (3) close observation of the patient's neurological function changes, such as state of consciousness, motor function and language ability; (4) recording of the disease development; and (5) assisting the patient in daily activities, such as washing, dressing and eating, to ensure that their basic living needs were met according to their specific circumstances, and developing an appropriate diet plan to ensure adequate nutritional support and promote rehabilitation.

Comfortable care programme: the interventions were explicitly designed based on Kolcaba's four comfort dimensions: (1) physical—personalized rehabilitation plan, nutritional support, vital signs monitoring, skin care, thrombosis prevention; (2) psychological—emotional support, psychological counseling, establishing nurse–patient relationship; (3) social—family communication and education; and (4) environmental—provision of a quiet and comfortable nursing space. Based on ensuring routine care, the following care methods were also included. (1) Based on the patient's self-care ability, a personalized rehabilitation plan was developed, including physical therapy, functional training and rehabilitation training to improve the patient's self-care ability and recovery function. (2) A comprehensive nutritional assessment was conducted to understand the nutritional status of patients; based on the evaluation results and the patient's taste preferences, a personalized diet plan was developed to ensure that patients received sufficient nutrients. (3) Patients were provided with emotional support and comfort and a good nurse–patient relationship was established, meaning patients' confidence and enthusiasm for treatment were enhanced, helping them to understand the development of relevant treatment programmes, disease progression, pipeline maintenance and the occurrence and treatment of adverse events. Psychological counseling and intervention were also provided to help patients cope with emotional and psychological distress during treatment. (4) Vital signs, including blood pressure, body temperature, pulse and respiration, were regularly observed to detect abnormalities promptly. Nursing measures, including turning over, bedding and skin care, were implemented to prevent pressure sores and other complications. (5) To prevent thrombosis, patients were encouraged to perform activities such as exercising their limbs and using anticoagulants on time. (6) Effective education and communication with the patient's family members were established, providing disease knowledge and nursing guidance, so that they could participate in the patient's care.

The intervention period of this study was 4 weeks. All patients completed the baseline assessment (T0) at the time of enrolment (before intervention). The first post-test (T1) was conducted within 24 h after the end of the intervention to evaluate the immediate effect. To evaluate the persistence of the short-term effect, the second post-test (T2) was conducted 1 month after the end of the intervention. The control group received the same assessment at the same time points. All assessments were completed by researchers who were unaware of the grouping situation.

### 2.3 Evaluation indicators

#### 2.3.1 National institutes of health stroke scale

The National Institutes of Health Stroke Scale (NIHSS) (15) contains 11 items to assess the patient's gaze, consciousness level, language ability, motor function, visual field and other neurological functions. Each item was scored according to the patient's performance, with a total score ranging from 0 to 42 points. The lower the score is, the better the neurological function, reflecting the severity of stroke and functional impairment. The NIHSS is widely used in many clinical studies, with good internal consistency (Cronbach's alpha >0.85) and reliability, and is considered to be an effective stroke severity assessment tool.

#### 2.3.2 Fugl-meyer assessment scale

The Fugl-Meyer Assessment (FMA) scale (16) primarily evaluates the motor function of the upper and lower limbs, including motor accuracy, coordination and strength, and is divided into two dimensions. The total score of the scale is 100 points; the highest score of the upper limbs was 66 points, and the highest score of the lower limbs was 34 points. The higher the score is, the better the motor function, which can reflect the patient's motor ability, recovery degree and daily activity ability. The FMA scale has high reliability (Cronbach's alpha >0.90) and validity in clinical practice.

#### 2.3.3 Activities of daily living scale

The Activities of Daily Living (ADL) scale (10) evaluates the patient's ability to perform basic activities in daily life and comprises 10 functions, including urination, toileting, dressing, eating and moving. The total score is 100 points, and each activity is scored according to the patient's self-care ability. The higher the score is, the better the patient's independent ability in daily life. The ADL scale has good internal consistency and test-retest reliability (Cronbach's alpha ≥0.87), and its effectiveness in reflecting the patient's living ability and self-care level accurately has been demonstrated in a number of studies.

#### 2.3.4 Newcastle satisfaction with nursing scale

The Newcastle Satisfaction with Nursing Scale (NSNS) (17) is used to evaluate patients' overall satisfaction with nursing services and covers many aspects, including quality of care, communication and meeting of their needs. The scale is usually calculated according to the patient's score for nursing service. The version used in this study contains a total of 19 items and uses the Likert 5-level scoring method (1 = very dissatisfied, 2 = dissatisfied, 3 = average, 4 = satisfied, 5 = very satisfied). The sum of the scores of all items is the total score, with the total score ranging from 19 to 95 points. The higher the score is, the higher the patient's satisfaction. The NSNS scale has shown good reliability (Cronbach's alpha ≥0.90) and validity in different nursing environments, thus effectively reflecting patients' feelings and evaluation of nursing services.

#### 2.3.5 Hamilton anxiety scale

The Hamilton Anxiety Scale (HAMA) (18) is used to evaluate the degree of anxiety of patients before and after nursing and includes various symptoms of psychological and physiological anxiety. The scale is divided into 14 items, each of which is scored according to the patient's condition. The total score range is usually 0–56 points. The higher the score is, the more obvious the anxiety symptoms. The HAMA has high reliability and validity, and Cronbach's alpha is typically >0.80. It is widely used in clinical practice and research to evaluate the degree of anxiety and is suitable for evaluating changes before and after treatment.

### 2.4 Statistical analysis

Statistical analysis was performed using SPSS 26.0 statistical software. The measurement data were expressed as mean ± standard deviation (x ± s), and the mean comparison between groups was performed using the *t*-test. The count data were expressed as frequency (*n*) or rate (%) using the chi-square (χ^2^) test. Effect sizes (Cohen's *d*) were calculated for inter-group comparisons of primary outcomes. A *p*-value of < 0.05 was considered statistically significant.

## 3 Results

### 3.1 Comparison of general data of patients

In this study, 120 elderly patients with acute cerebral infarction were randomly divided into an intervention group and a control group. There were 34 men and 23 women in the intervention group, with an average age of 65.46 ± 3.77 years, and there were 38 men and 20 women in the control group, with an average age of 66.34 ± 4.16 years. There was no significant difference in age, sex ratio, BMI, marital status and basic diseases between the two groups (*p* > 0.05), as shown in Table 1.

**Table 1:** Comparison of general data of patients.

Variable	Experimental group (*n* = 57)	Control group (*n* = 58)	*t*/χ^2^	*p-*value
Mean age	65.46 ± 3.77	66.34 ± 4.16	−1.199	0.233
Gender			0.423	0.516
Male	34	38		
Female	23	20		
BMI index **(**kg/m^2^**)**	23.89 ± 1.43	23.97 ± 1.76	−0.268	0.790
Marital status			0.078	0.780
Non-married	12	11		
Married	45	47		
Underlying			0.091	0.763
diseases				
Yes	48	50		
No	9	8		

### 3.2 Comparison of national institutes of health stroke scale scores between the two groups

Before the intervention, the NIHSS score of the experimental group was 21.33 ± 2.75, and that of the control group was 21.59 ± 3.13. There was no significant difference in the scores between the two groups before intervention (*t* = −0.459, *p* = 0.647), indicating that the severity of the two groups before intervention was similar. Following intervention, the NIHSS score of the experimental group was reduced to 6.82 ± 1.02, and the score of the control group was reduced to 11.24 ± 1.79. Statistical analysis showed that the difference in scores between the two groups after intervention was statistically significant (*t* = −16.220, *p* < 0.001), with a large effect size (Cohen's *d* = 2.97). This indicated that the nursing measures in the experimental group had a significant effect on reducing the severity of stroke, as shown in Table 2.

**Table 2:** Comparison of NIHSS scores within and between groups before and after intervention.

Group	Number	Before intervention	After intervention	*t-*value	*p-*value
Experimental group	57	21.33 ± 2.75	6.82 ± 1.02	39.986	< 0.001
Control group	58	21.59 ± 3.13	11.24 ± 1.79	21.589	< 0.001
*t-*value		−0.459	−16.220		
*p-*value		0.647	< 0.001		

### 3.3 Comparison of fugl-meyer assessment scores between the two groups

Before the intervention, the FMA score of the experimental group was 41.86 ± 5.51, and that of the control group was 42.55 ± 6.38. There was no significant difference in the scores between the two groups before the intervention (*t* = −0.622, *p* = 0.535), indicating that the functional status of the two groups was similar before the intervention. Following intervention, the FMA score of the experimental group was increased to 75.95 ± 7.71, and the score of the control group was increased to 63.34 ± 6.59. The statistical analysis showed that there was a statistically significant difference in the scores between the two groups after the intervention (*t* = 9.433, *p* < 0.001), with a large effect size (Cohen's *d* = 1.78). This indicated that the intervention measures adopted in the experimental group effectively promoted the recovery of functional movement and improved the daily living ability of the patients, as shown in Table 3.

**Table 3:** Comparison of FMA scores within and between groups before and after intervention.

Group	Number	Before intervention	After intervention	*t-*value	*p-*value
Experimental group	57	41.86 ± 5.51	75.95 ± 7.71	−26.173	< 0.001
Control group	58	42.55 ± 6.38	63.34 ± 6.59	−16.696	< 0.001
*t-*value		−0.622	9.433		
*p-*value		0.535	< 0.001		

### 3.4 Comparison of activities of daily living scores between the two groups

Before the intervention, the ADL score of the experimental group was 32.00 ± 3.88, and that of the control group was 32.36 ± 4.26. There was no significant difference in the scores between the two groups before the intervention (*t* = −0.476, *p* = 0.635), indicating that at the beginning of the intervention, the daily living ability of the two groups was similar. Following intervention, the ADL score of the experimental group was increased to 69.61 ± 6.98, and the score of the control group was increased to 59.81 ± 4.93. Statistical analysis showed that the difference in scores between the two groups after intervention was statistically significant (*t* = 8.713, *p* < 0.001), with a large effect size (Cohen's *d* = 1.67). This indicated that the nursing measures applied to the experimental group effectively improved the patient's daily living ability, promoted functional recovery and improved the patient's quality of life, as shown in Table 4.

**Table 4:** Comparison of ADL scores within and between groups before and after intervention.

Group	Number	Before intervention	After intervention	*t-*value	*p-*value
Experimental group	57	32.00 ± 3.88	69.61 ± 6.98	−36.068	< 0.001
Control group	58	32.36 ± 4.26	59.81 ± 4.93	−30.761	< 0.001
*t-*value		−0.476	8.713		
*p-*value		0.635	< 0.001		

### 3.5 Comparison of newcastle satisfaction with nursing scale score and hamilton anxiety scale score between the two groups

The NSNS score of the experimental group was 5.05 ± 0.35, whereas that of the control group was 3.07 ± 0.26. Statistical analysis showed that the difference in NSNS scores was significant (*t* = 34.782, *p* < 0.001), with a large effect size (Cohen's *d* = 6.59), indicating that the neuropsychological status assessment scores of the experimental group were significantly better than those of the control group. The HAMA score of the experimental group was 7.84 ± 2.80, and that of the control group was 14.48 ± 3.50. The analysis showed that the HAMA score difference was also significant (*t* = −11.229, *p* < 0.001), with a large effect size (Cohen's *d* = 2.15), indicating that the anxiety level of the experimental group was significantly lower than that of the control group, as shown in Table 5.

**Table 5:** Comparison of NSNS and HAMA scores between groups after intervention.

Group	Number	NSNS	HAMA
Experimental group	57	5.05 ± 0.35	7.84 ± 2.80
Control group	58	3.07 ± 0.26	14.48 ± 3.50
*t-*value		34.782	−11.229
*p-*value		< 0.001	< 0.001

## 4 Discussion

Our findings are consistent with several recent randomized controlled trials (RCTs) that have applied holistic or comfort-based interventions in stroke care. For example, an RCT by Li et al. (19) demonstrated that integrated comfort care significantly improved the psychological wellbeing of patients post-stroke, which aligns with our HAMA results (20). Elderly patients with acute cerebral infarction often face complex rehabilitation challenges due to physiological, psychological and social environment factors. Therefore, it is particularly important to provide individualized care for these patients. Comfort care theory is a nursing theory proposed by Dr Katharine Kolcaba in the 1990s, which aims to improve the overall health and wellbeing of patients by providing a comprehensive comfort experience (21). The theory emphasizes that “comfort” means not only physical comfort but also psychological, social and spiritual comfort. Comfort is regarded as a multidimensional concept, including physical, psychological, social and environmental considerations. Physical comfort refers to a state of physical pleasure; psychological comfort focuses on the patient's emotional and mental health; social comfort addresses the patient's interpersonal relationships and social support; environmental comfort is related to the comfort and safety provided by the patient's environment (22). The primary goal of comfort care is to promote health and recovery by meeting the comfort needs of patients. Nursing workers should strive to identify patients' needs and take appropriate measures to improve their comfort. This theory has a wide range of applications in clinical nursing practice and provides a systematic nursing framework for nurses to better meet patients' needs.

The results of this study reveal that the experimental group had a significant improvement in functional exercise recovery, daily living ability and psychological state, indicating that the nursing intervention measures adopted effectively promoted the comprehensive rehabilitation of patients with stroke. This result is consistent with the research results in the relevant literature, emphasizing the importance of active intervention for patients with stroke on their functional and mental health. In terms of functional exercise recovery, the FMA score of the experimental group was significantly higher than that of the control group, which may be attributed to a more systematic and personalized rehabilitation plan. By formulating individualized nursing strategies, more accurate interventions can be provided according to the needs of different patients, thus effectively improving their exercise and self-management abilities. In the improvement of daily living ability, the ADL score of the experimental group was significantly improved, indicating that nursing intervention not only enhanced the physical function of patients but also improved their ability to participate in daily activities. This change helps patients rebuild their confidence and independence, thus promoting a better quality of life. In addition, the experimental group showed lower anxiety levels and better neuropsychiatric status in terms of psychological status, indicating that nursing measures played an important role in psychological support and emotional care. Anxiety is a common complication of patients with stroke, affecting their recovery process. Therefore, the implementation of comprehensive nursing measures can help to reduce the psychological burden of patients and provide a positive psychological environment, thus promoting overall rehabilitation. In conclusion, the results support the necessity and effectiveness of comprehensive nursing intervention for patients with stroke. In future studies, the specific mechanisms of different nursing measures can be explored, and the long-term effects can be evaluated to provide a more solid theoretical basis for clinical practice.

Although this study has drawn positive conclusions, there are still some limitations that need attention. First, the sample size is relatively small, which may affect the generalization of the results. Future studies may consider expanding the sample size to enhance the reliability of statistics and the general applicability of the results. Second, the observation period of this study was short, and the long-term effect of nursing intervention was not evaluated. Therefore, it is recommended that follow-up studies use a longer follow-up time to observe the patient's continuous functional recovery and quality of life changes. In addition, there may be differences in baseline characteristics between the experimental group and the control group, which may have an impact on the results. Future research should be conducted on the basis of random grouping to enhance the reliability of the results.

Nursing intervention for patients with stroke has a wide range of clinical benefits. Future research can compare the effects of different types of nursing intervention (e.g., physical therapy, psychological counseling, nutritional support) to find the best combination of interventions to achieve a more comprehensive improvement effect. Based on the specific conditions of patients, we should develop individualized nursing programmes, analyse the differences in response of different patients and provide more targeted nursing methods for clinical practice. We should endeavor to study the independent effect of psychological support on the rehabilitation of patients with stroke and explore modes of combining psychological intervention with medical nursing to improve the overall therapeutic effect. Furthermore, long-term follow-up surveys should be established to evaluate the impact of nursing intervention on patients' long-term health and quality of life and establish a corresponding evaluation mechanism to provide a basis for future clinical decision-making. By actively exploring these directions, future research will further enrich the theoretical basis of nursing intervention for patients with stroke and provide a more comprehensive solution for improving their treatment effect and quality of life.

## 5 Conclusion

The results of this study indicate that the experimental group was significantly better than the control group in functional exercise recovery (FMA score), daily living ability (ADL score) and psychological state (NSNS and HAMA scores) following specific nursing intervention. The results reveal that the nursing intervention measures in the experimental group had significant effects in improving the functional recovery, daily living ability and psychological state of patients after stroke, which suggests that the measures have important clinical application value. It is recommended to promote the application of this intervention in the nursing process of patients post-stroke to improve their overall treatment outcome and quality of life.

## Data Availability

The original contributions presented in the study are included in the article/supplementary material, further inquiries can be directed to the corresponding authors.
